# Severe Asthma in the Era of Biologics: Continuous Challenges

**DOI:** 10.3390/jcm12113857

**Published:** 2023-06-05

**Authors:** Pinelopi Schoini, Petros Bakakos, Stelios Loukides

**Affiliations:** 1 4th Respiratory Department, Athens Chest Hospital Sotiria, 11527 Athens, Greece; pinelopi_schoini@hotmail.com; 2 1st Respiratory Medicine Department, Athens Chest Hospital Sotiria, Medical School, National and Kapodistrian University of Athens, 12462 Athens, Greece; petros44@hotmail.com; 3 2nd Respiratory Medicine Department, Attiko University Hospital, Medical School, National and Kapodistrian University of Athens, 12462 Athens, Greece

Asthma is a heterogenous disease characterized by different phenotypes and endotypes. Severe asthma is a unique phenotype of the disease characterized by two major endotyping mechanisms—the high T2 process and the low T2 process. The definition of severe asthma is mainly based on the high requirements of treatment regimens. In the last 20 years, biologic agents have had a central role in targeting the previously refractory T2 high process, with many randomized controlled trials (RCTs) clearly verifying both safety and efficacy [1]. Despite their effectiveness in different aspects of disease assessment there are many challenges which need to be either addressed or resolved. These aspects are mainly attributed to the presence of co-morbidities, long-term effects of treatment, and baseline characteristics of the selected patients, as well as to additive treatment strategies.

An important issue in severe asthma is the presence of bronchiectasis. In a recently published study, the presence of high blood eosinophilia in patients with severe asthma and bronchiectasis was mainly attributed to the different localization of bronchiectasis [2]. The above finding may indirectly reveal aspects of treatment interventions, such as an inflammatory profile sensitive to high T2 targeting interventions; however, simultaneously, a question arises: In a disease where neutrophils are mainly involved, how effective are treatments which eliminate the refractory high T2 process? In a population characterized by severe asthma with co-existing bronchiectasis, the presence of leukotriene receptor antagonists (LTRAs) in treatment strategies may influence both the T2 pathway and the neutrophilic component of the disease [3]. The above possible beneficial effects resulted in lower fractional exhaled nitric oxide (FeNO) values as well as less use of health services in a 6-month assessment period.

Randomized control trials (RCTs) are critical in the process of drug authorizations. In severe asthma, many RCTs have proven the efficacy and the safety of different biologic agents. The population of those trials is high-T2-based. However, considering the different biologic treatments, we have to point out that we have a significant overlap in terms of the underlying mechanism and its corresponding phenotype. We have to discriminate the official indications for each biologic agent from the aspects that drive their selection. Considering the primary outcomes of the authorization studies, all of them are focused on the rate of exacerbation, since the latest is a critical aspect of disease severity. Furthermore, the steroid-sparing effect was one of the primary outcomes, which was addressed in pilot authorization studies. As secondary outcomes, RCTs were used to analyze several aspects, such as lung function, quality of life, and control of the disease [4].

Additionally, but to a lesser extent, some biomarkers were used either as predictors of treatment responses or to confirm the benefits of T2 process elimination. Interestingly, all biologic agents were effective in reducing exacerbation rates and in improving asthma control. However, some differences existed in terms of the other targeting aspects. Benralizumab, mepolizumab, and dupilumab have a proven efficacy as steroid-sparing agents. On the other hand, the above benefit has not been seen for tezepelumab and omalizumab. A study has been performed on mepolizumab, focusing on quality of life as a primary outcome with positive results. Dupilumab showed a greater benefit in lung function improvement, indicating that its mechanism—and particularly the IL-13 inhibition—may alter the airway’s smooth muscle contraction in a better way. Co-morbidities have a multidimensional role. They are part of the predictive process but at the same time they are disease-centered outcomes. What fundamentally guides the selection process is a multi-factorial approach, which involves a patient’s clinical history, predominant endotype, comorbidities, and disease onset. Focusing on blood eosinophils, it is important to clarify that are both selective parameters but also predictive ones [4].

Apart from the RCTs, real life provided us with important information in terms of biologics use in clinical practice. In the majority of the real-life studies, the benefits are greater than those observed in RCTs. A typical paradigm among many others is a benralizumab study where 2 years treatment in severe asthma patients taking maintenance treatment with OCS showed a steroid-sparing effect in approximately 60% of the patients, while a dose reduction existed in 85% of the study population [5]. Meanwhile, 85% of patients had their asthma well controlled (ACT score > 20) and had no exacerbations, while 42% normalized their lung function. An important and simultaneously provocative question that is arising is how long we have to administer the biologic treatment—for life or by using booster administrations? A study was conducted that compared continuous vs. boost omalizumab, and it found that continuous use was superior, while the boost approach worsened the long-term beneficial outcomes [6].

Without a doubt, a biologic agent either affects the natural course of the disease and/or eliminates the harmful effects of many parameters, leading to alterations of disease status. The arising question is whether it could also modify the disease. This needs inflammatory assessment and clear messages about treatment de-escalation. The future consists of many challenges. The first and most important is whether treatment with biologics may lead to disease remission. However, the definition of remission still warrants further clarifications. Additionally, we have to discriminate the term response from the term remission and to state that there are two parallel and not competitive procedures. Finally, we have to understand that the selection of biologics in the majority of cases represents an overlapping procedure, and the first is not always better. On the other hand, if the first choice failed to confirm our expectations, then the switch process with proper selection is the alternative option.
